# Case Report: Duchenne muscular dystrophy–associated cardiomyopathy presenting as adolescent nonischemic heart failure—from red-flag clues to genetic confirmation and multisystem management

**DOI:** 10.3389/fcvm.2026.1782721

**Published:** 2026-09-24

**Authors:** Wenxuan Li, Yangang Wang

**Affiliations:** The Affiliated Hospital of Qingdao University, Qingdao, China

**Keywords:** adolescent nonischemic heart failure, DMD-associated cardiomyopathy, Duchenne muscular dystrophy, genetic confirmation, multisystem management

## Abstract

**Background:**

Duchenne muscular dystrophy (DMD) can involve the myocardium and progress to cardiomyopathy and heart failure; however, diagnosis is frequently delayed because initial clinical manifestations are often atypical. In adolescent males presenting with nonischemic cardiomyopathy of unclear etiology, early integration of a coherent evidence chain spanning neuromuscular clues, multimodality imaging, and genetic testing is pivotal for etiologic clarification and optimization of long-term management.

**Case summary:**

The patient was a 17-year-old adolescent male who presented repeatedly with chest tightness, fatigue, and reduced exercise tolerance. During the first hospitalization in September 2025, evaluation revealed left atrial and left ventricular enlargement with impaired systolic function (LVEF ∼44%). Laboratory testing showed marked hyperCKemia (CK 1,451 U/L), mildly elevated hs-cTnT (0.033 μg/L), and concomitant transaminase elevation. Coronary computed tomography angiography demonstrated no significant stenosis, with only a superficial myocardial bridge in the mid–left anterior descending artery. According to the original clinical cardiac magnetic resonance report, multifocal patchy late gadolinium enhancement was described predominantly in a subepicardial distribution, together with a trabeculated/noncompaction-like left ventricular morphology. During the second hospitalization in November 2025, focused neuromuscular assessment uncovered a long-standing progressive proximal weakness (EMG-supported), linking the cardiac phenotype to dystrophinopathy (positive Gower's sign, calf pseudohypertrophy, and waddling gait), and electromyography supported a myopathic process; transthoracic echocardiography showed improvement of LVEF to 50%. At the third hospitalization in December 2025, the dominant issues were hyperglycemia with proteinuria (HbA1c 7.7% and UACR ∼389 mg/g), while echocardiography documented further recovery of LVEF to 55%. Genetic testing identified a DMD variant (NM_004006.3:c.1351G>T, p.Asp451Tyr), and Sanger sequencing confirmed hemizygosity in the proband and heterozygosity in his mother. Taken together, the convergent clinical, imaging, electrophysiologic, and genetic findings established the diagnosis of DMD-associated cardiomyopathy. Under guideline-directed heart failure therapy and multidisciplinary care, the patient demonstrated longitudinal improvement in cardiac function (LVEF ∼44% → 50% → 55%).

**Conclusion:**

In adolescent males with nonischemic cardiomyopathy and/or heart failure of unclear etiology—particularly when accompanied by hyperCKemia or suspected myogenic transaminase elevation—dystrophinopathy should be considered early in the differential diagnosis. A diagnostic “closed loop” can be achieved by integrating cardiac phenotyping, neuromuscular examination, electromyography, family history, and confirmatory genetic testing, while guideline-directed heart failure therapy and proactive family-based care (including cardiac surveillance of female carriers) should be initiated in parallel to improve long-term outcomes.

## 1 Introduction

Duchenne muscular dystrophy (Duchenne muscular dystrophy, DMD) is an X-linked recessive, progressive muscle-wasting disorder caused by pathogenic variants in the DMD gene, leading to loss of dystrophin expression or function. This molecular defect drives relentless injury to both skeletal and cardiac muscle and is ultimately associated with poor outcomes, most commonly due to cardiopulmonary complications (1). With advances in ventilatory support and comprehensive multidisciplinary care, survival has improved, and cardiomyopathy has consequently emerged as a principal determinant of both longevity and quality of life in this population (1, 2).

For the early recognition and risk stratification of DMD-associated cardiomyopathy, cardiac magnetic resonance (CMR) offers more sensitive markers of myocardial injury than conventional echocardiography. Even when left ventricular ejection fraction remains preserved, late gadolinium enhancement (late gadolinium enhancement, LGE) can reveal the burden of myocardial fibrosis and has been linked to increasing age and subsequent deterioration in left ventricular systolic function (3, 4). Moreover, CMR-derived strain metrics may capture evolving myocardial involvement before overt changes in traditional indices become apparent, thereby refining follow-up strategies and helping to define the optimal window for intervention (5).

From a therapeutic standpoint, current consensus statements underscore the importance of initiating and optimizing cardioprotective therapy as early as possible, with the goal of slowing fibrotic progression and preserving left ventricular function (6). Prior studies suggest that neurohormonal antagonism—most notably β-blockers and ACE inhibitors/angiotensin receptor blockers (ACEI/ARB)—may confer clinically meaningful benefit in DMD-related cardiac involvement (7), and randomized controlled data further support the addition of a mineralocorticoid receptor antagonist (e.g., eplerenone) during the “early cardiomyopathy” stage to attenuate functional decline (8). At the same time, DMD patients may experience episodes of acute myocardial injury or myocarditis-like exacerbations that can mimic acute coronary syndromes, thereby increasing diagnostic uncertainty and management complexity (9, 10). Against this background, we present the current case using a timeline-based narrative combined with longitudinal comparisons of key indices, highlighting the real-world evolution of DMD-associated myocardial involvement and the pivotal diagnostic and therapeutic decision points, with the aim of offering a reproducible clinical approach for earlier recognition and risk-stratified management in similar patients.

## 2 Case features

### 2.1 Timeline overview

In September 2025, the patient was first hospitalized for chest tightness with reduced exercise tolerance (8-day stay). Echocardiography demonstrated left-sided cardiac enlargement and impaired left ventricular systolic function (LVEF ∼44%). During the same admission, coronary CTA excluded significant coronary stenosis, while the original clinical CMR report described multifocal LGE predominantly in a subepicardial distribution and a trabeculated/noncompaction-like left ventricular morphology. In November 2025, he was readmitted for progressive limb weakness with intermittent chest tightness and dyspnea (8-day stay). Physical examination identified a positive Gower's sign, calf pseudohypertrophy, and a waddling gait; electromyography supported a myopathic process. Repeat echocardiography showed improvement of LVEF to ∼50%. In December 2025, he was hospitalized a third time because of hyperglycemia accompanied by proteinuria (13-day stay). While metabolic and renal involvement were evaluated and therapies adjusted, the previously established guideline-based heart failure regimen was maintained. Echocardiography during this admission documented further improvement in LVEF to ∼55%, alongside additional symptomatic improvement compared with prior presentations (Figure 1).

**Figure 1:**
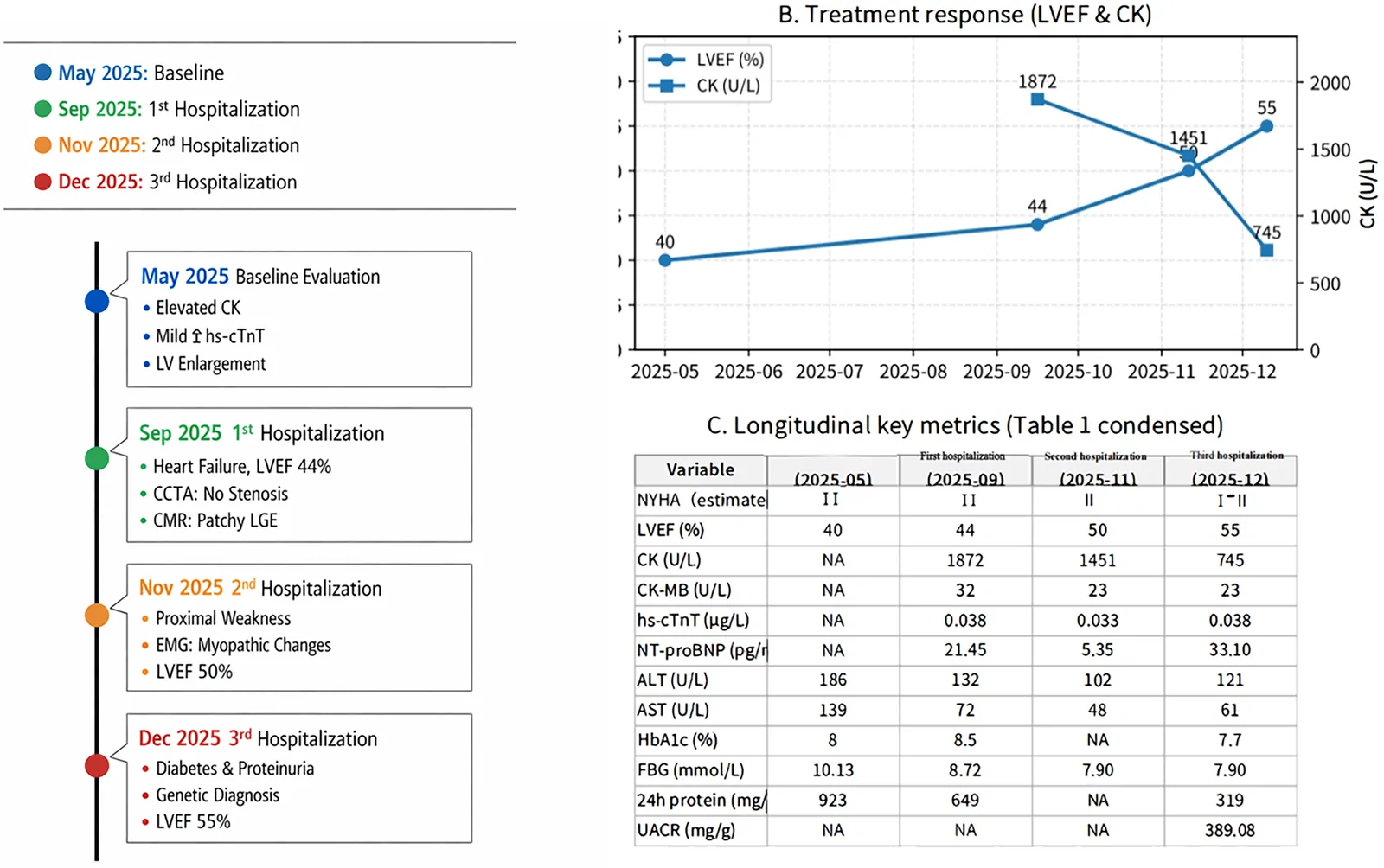
Diagnostic timeline and treatment response (text-derived; values from provided Table 1).

### 2.2 First hospitalization

The patient was a 17-year-old adolescent male with an approximately 2-year history of recurrent chest tightness, with a marked worsening over the preceding 5 months accompanied by fatigue and reduced exercise tolerance (notably exertional difficulty when walking uphill). He also reported occasional palpitations and intermittent chest pain. At presentation, his overall clinical picture was dominated by mild-to-moderate exertional symptoms consistent with a heart failure phenotype; based on the history, his functional status was estimated as New York Heart Association (NYHA) class II (estimated). During this admission, the principal clinical problem was approached as unexplained left ventricular systolic dysfunction with a dilated cardiomyopathy–like phenotype, prompting systematic etiologic evaluation and initiation of appropriate management.

Following admission, a comprehensive diagnostic work-up was completed with the following key findings. (1) ECG (Figure 2) showed sinus rhythm with a heart rate of ∼99 bpm; PR ∼122 ms, QRS ∼101 ms, and QT/QTc ∼342/439 ms. (2) Transthoracic echocardiography (Figure 3) demonstrated left atrial and left ventricular enlargement with globally reduced wall motion and impaired systolic function, with an LVEF of 44% (EDV ∼135 mL). Right ventricular systolic function was preserved (TAPSE ∼2.2 cm), the estimated pulmonary artery systolic pressure was ∼23 mmHg, and there was only mild mitral and tricuspid regurgitation. The echocardiographic impression was “left atrial enlargement, left ventricular enlargement, and reduced left ventricular systolic function”. (3) Biomarkers: during hospitalization, the muscle enzyme profile revealed elevated CK (1,451 U/L) and myoglobin (205 μg/L), while hs-cTnT was mildly increased (0.033 μg/L). Transaminases were also elevated; in the context of concomitant hyperCKemia, a myogenic source of aminotransferase elevation warranted consideration. (4) Coronary CTA showed no significant coronary stenosis or anatomic anomaly, with a right-dominant system and a superficial myocardial bridge in the mid–left anterior descending artery. Collectively, these findings argued against ischemic cardiomyopathy and supported a nonischemic etiologic work-up. (5) Cardiac magnetic resonance imaging was performed during the first hospitalization on 21 September 2025. According to the original clinical CMR report, left ventricular systolic function was impaired, consistent with the echocardiographic LVEF of approximately 44%, and multifocal patchy late gadolinium enhancement was described predominantly in a subepicardial distribution. The report also noted a trabeculated/noncompaction-like left ventricular morphology. These report-based findings supported a nonischemic myocardial disease process but were interpreted together with the clinical, biochemical, neuromuscular, electrophysiologic, and subsequent genetic findings rather than as a standalone diagnostic criterion. The scanner vendor, field strength, parametric mapping values, and strain data could not be retrospectively retrieved from the available archived clinical records.

**Figure 2:**
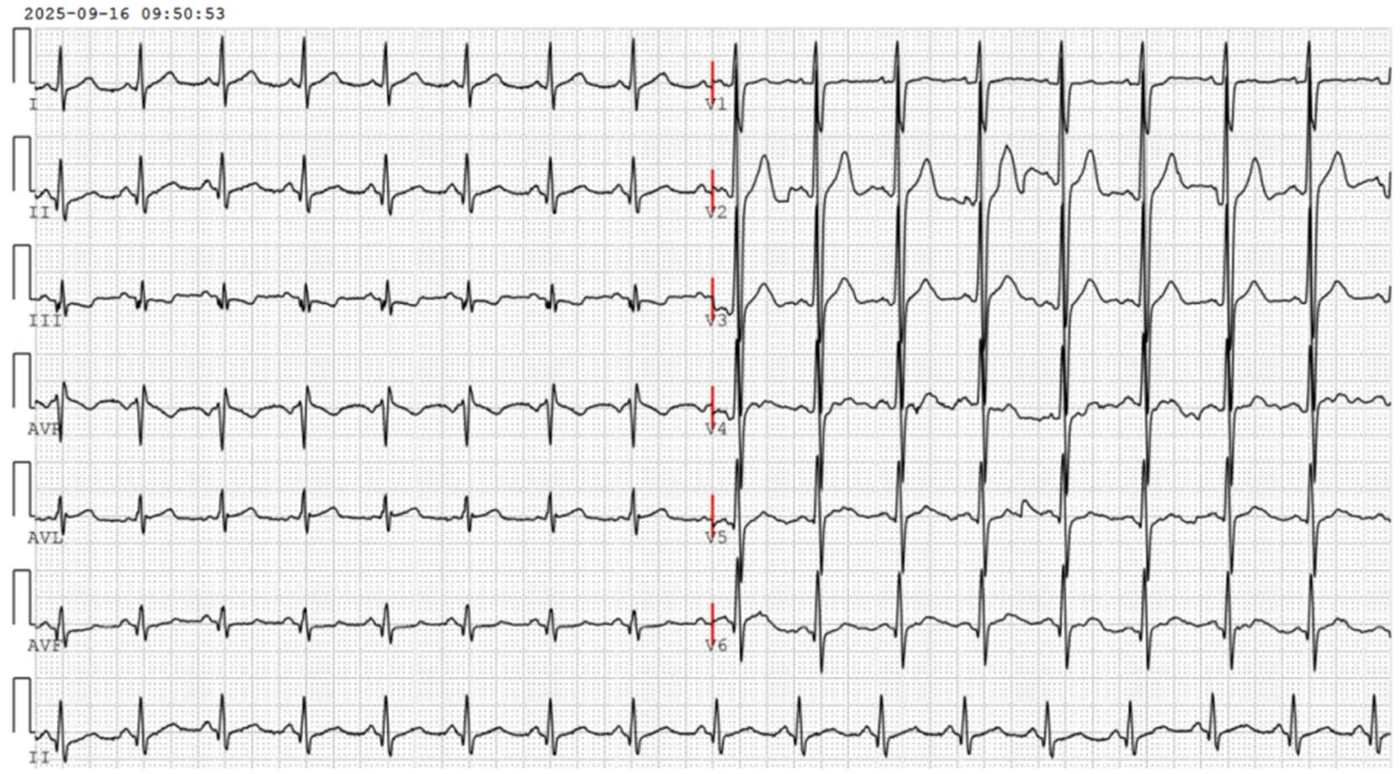
Electrocardiogram during the first hospitalization.

**Figure 3:**
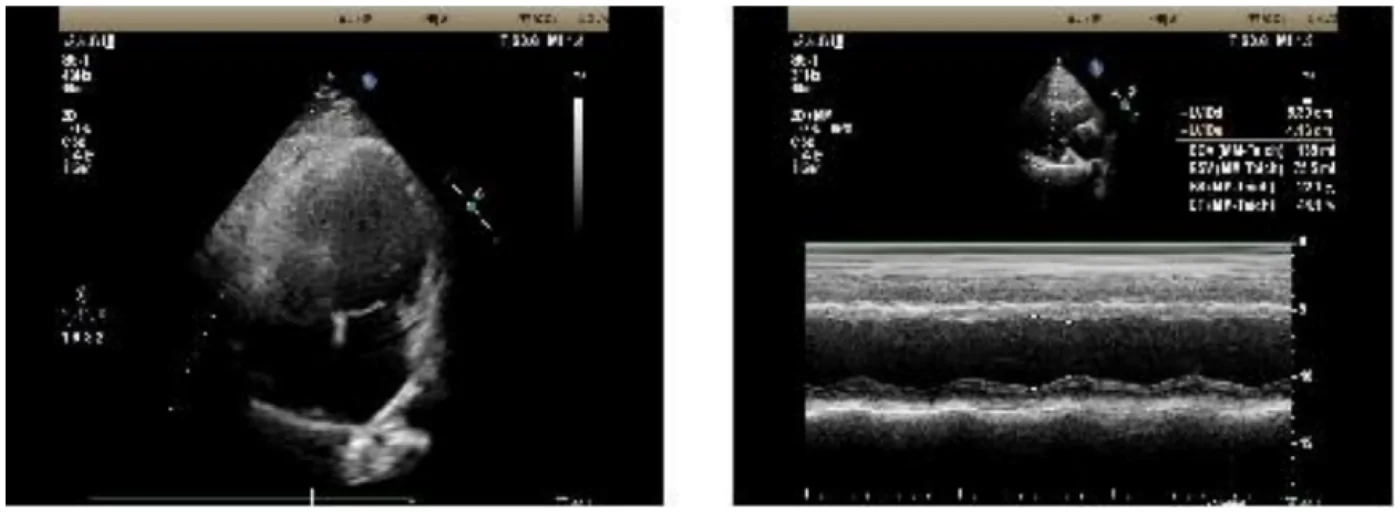
Transthoracic echocardiography during the first hospitalization.

Within the diagnostic and management framework for adolescent nonischemic cardiomyopathy/heart failure, the patient received a comprehensive regimen centered on guideline-directed medical therapy for heart failure. Neurohormonal blockade was instituted with an ARNI-based strategy, complemented by a β-blocker initiated and up-titrated in a stepwise manner. A mineralocorticoid receptor antagonist (e.g., spironolactone) and a loop diuretic (e.g., furosemide) were used for volume management, and an SGLT2 inhibitor (e.g., dapagliflozin) was incorporated as part of the long-term heart failure disease–modifying backbone. Additional agents—including digoxin and trimetazidine—were administered for symptomatic relief and metabolic support. In parallel, etiologic screening was pursued and a structured follow-up pathway was established, leaving a deliberate “diagnostic interface” for subsequent refinement of a potential myopathy-related mechanism of myocardial involvement.

After this treatment course, chest tightness and exercise tolerance improved compared with admission, and vital signs remained stable. He was discharged after an 8-day hospitalization. At discharge, long-term heart failure maintenance therapy was continued, anchored by ARNI/β-blocker/MRA/SGLT2i with diuretics as needed, and outpatient cardiology follow-up was arranged together with ongoing etiologic evaluation (including intensified screening for neuromuscular clues and genetic assessment). This strategy provided continuity for the subsequent readmissions and ultimately facilitated establishment of the definitive diagnosis.

At the time of this first admission, the patient had not been diagnosed with DMD. Notably, the admission record already documented a maternal family history of DMD (an affected uncle) and a history suggestive of progressive myopathy/weakness, but these clues were not yet integrated into a unified etiologic hypothesis during the initial cardiology-focused evaluation. In retrospect, earlier synthesis of these “red flags” together with the markedly elevated CK and aminotransferases could have prompted earlier neuromuscular work-up and genetic testing.

### 2.3 Second hospitalization

The patient was readmitted on 2025-11-11 (8-day stay), with the chief complaint centered on limb weakness of more than 4 years’ duration, accompanied by intermittent chest tightness and dyspnea. Importantly, on detailed history-taking, the patient reported progressive proximal limb weakness for more than 4 years, which predated his cardiopulmonary symptoms and was only recognized as a neuromuscular clue during this admission. Building on the previously documented family history and progressive weakness, targeted neuromuscular examination during this admission identified classic dystrophinopathy signs (Gowers’ sign, calf pseudohypertrophy, and waddling gait), and EMG supported a myopathic pattern—thereby accelerating etiologic clarification and subsequent genetic confirmation. Against the background of adolescent nonischemic cardiomyopathy/heart failure, the pivotal inflection point of this admission was the deliberate expansion beyond a cardiology-only follow-up framework: targeted neuromuscular history-taking and focused physical examination elicited clinical features consistent with progressive proximal muscle weakness (e.g., difficulty rising from a seated position and climbing stairs). This reframed the working differential by bringing “cardiac involvement + skeletal muscle involvement” into a single unifying etiologic chain. Based on his symptom profile, functional status was estimated as NYHA class II (estimated).

Further investigations during this admission included: (1) ECG, which demonstrated sinus rhythm with a heart rate of ∼105 bpm. The ECG report described sinus tachycardia and suggested changes such as abnormal Q waves in leads III and aVF, warranting interpretation in the clinical context and in comparison with prior tracings. (2) Echocardiography showed improved left ventricular systolic function relative to the prior admission, with an LVEF of ∼50%.

Evidence chain for myopathic injury and interpretation of transaminase elevation. Building on the previously documented marked hyperCKemia and elevated aminotransferases, this admission treated the “muscle enzyme/transaminase pattern” as an actionable diagnostic clue, prompting systematic neuromuscular evaluation. In such patients, elevations in ALT/AST should raise suspicion for a substantive contribution from skeletal muscle injury rather than being attributed solely to primary liver disease, as a liver-centered pathway may delay recognition and referral for muscular dystrophy (1). Electromyography (EMG) supported a myopathic process, consistent with a progressive muscular dystrophy phenotype, and provided electrophysiologic corroboration for non-neurogenic weakness.

Given that the established heart failure regimen appeared effective—reflected by improvement in LVEF—this admission continued and optimized long-term guideline-based therapy centered on ARNI, a β-blocker, SGLT2i, and MRA, with routine monitoring of diuretic needs and electrolytes according to comorbidity status. More importantly, as neuromuscular clues became explicit, a dystrophinopathy-oriented management pathway was initiated/strengthened during hospitalization, including corticosteroid therapy (prednisone) with concomitant monitoring and preventive measures for anticipated adverse effects (e.g., bone protection and metabolic surveillance). In parallel, confirmatory genetic testing and family evaluation were initiated.

Following these interventions, symptoms such as chest tightness and dyspnea improved, and overall clinical status remained stable. The patient was discharged after an 8-day hospitalization. The discharge plan emphasized continuation of long-term guideline-directed heart failure therapy; completion of genetic testing and family-based validation; and enrollment into a coordinated neuromuscular–cardiac follow-up pathway to monitor myocardial fibrosis progression, arrhythmic risk, and the broader multisystem burden requiring longitudinal management.

### 2.4 Third hospitalization

The patient was admitted for the third time on 2025-12-10, with hyperglycemia and proteinuria of approximately 3 months’ duration. The primary purpose of this admission was further evaluation and treatment of presumed type 2 diabetes mellitus with renal involvement/proteinuria. DMD-related myopathy with cardiac involvement had already been established on the basis of prior evidence (heart failure presentation and myocardial injury phenotype), and heart failure–related symptoms were mild at this admission. Physical examination showed a BMI of ∼31.8 kg/m^2^ without clinically apparent edema. Functional class was estimated as NYHA class I–II (estimated) based on the clinical course and examination findings.

A repeat work-up during this admission yielded the following findings. (1) ECG demonstrated sinus rhythm/sinus arrhythmia, with reported abnormal Q waves in leads III and aVF and T-wave changes in leads I and aVL. These findings were considered potential clues to myocardial involvement or nonspecific repolarization abnormalities and were interpreted in the context of the patient's previously documented cardiomyopathy. (2) Echocardiography indicated further improvement in left ventricular systolic function, with an LVEF of ∼55%. (3) Markers of myocardial injury and volume status (longitudinal tracking): NT-proBNP was 33.1 pg/mL (reference range: <125 pg/mL for non-acute settings; <300 pg/mL is commonly used to rule out acute heart failure), which was not suggestive of significant volume overload and was consistent with the patient's improving symptoms and echocardiographic recovery (LVEF ∼55%). We interpreted this finding in the context of obesity (BMI ∼31.8 kg/m^2^), which may lower natriuretic peptide levels, and emphasized that longitudinal clinical status and imaging remained the primary indicators of improvement. The muscle enzyme profile remained abnormal, with CK ∼745 U/L and CK-MB ∼23 U/L. (4) Glycemic and renal indices (pre-admission/in-hospital testing): HbA1c was 7.7% and fasting glucose was ∼7.9 mmol/L. Urinalysis indicated proteinuria (previously “protein 2+”), supported by quantitative assessment with a urinary albumin-to-creatinine ratio (ACR) of ∼389 mg/g. Twenty-four-hour urinary protein excretion was ∼0.319 g/24 h. Earlier evaluation documented proteinuria (dipstick 2+), and thus the 24-hour quantification was interpreted as confirming persistent but relatively modest proteinuria. Renal function was preserved, with serum creatinine ∼46 μmol/L. Aminotransferases remained elevated (ALT ∼121 U/L and AST ∼61 U/L).

Therapeutic priorities during this admission focused on the metabolic–renal axis while maintaining a stable cardioprotective regimen and ongoing dystrophinopathy-oriented care. Heart failure/myocardial protection (maintenance of prior regimen): ARNI (sacubitril/valsartan), a β-blocker (metoprolol succinate), SGLT2i (dapagliflozin), and an MRA (e.g., spironolactone, with concomitant and/or alternative use of finerenone for proteinuria/renal protection) were continued. According to the clinical course documentation, there was no evidence necessitating intensified diuresis or acute decompensated heart failure rescue therapy during this hospitalization. This long-term cardioprotective strategy is consistent with the management principles of DMD-associated cardiomyopathy (6, 7). Glycemic control and weight management: in addition to prior oral glucose-lowering therapy, semaglutide was initiated at 0.25 mg once weekly by subcutaneous injection, accompanied by lifestyle interventions.

Overall, the patient remained clinically stable during hospitalization, with heart failure symptoms further alleviated or not exacerbated compared with the pre-admission state. After therapeutic adjustment focused on glycemic control and proteinuria management, he was discharged after a 13-day stay. Post-discharge follow-up was advised to include repeat echocardiography to reassess LVEF, ambulatory ECG monitoring (Holter), and repeat CMR when indicated for risk stratification. Because pulmonary function testing and standardized neuromuscular functional scales were not assessed, the follow-up plan should explicitly incorporate timely pulmonary function evaluation, with annual reassessment thereafter to support long-term outcome optimization.

Building on the established evidence chain—including persistent hyperCKemia and aminotransferase abnormalities, characteristic neuromuscular findings, a myopathic pattern on electromyography, cardiac dysfunction on echocardiography, and the documented family history—this admission completed molecular confirmation, and persistent hyperCKemia with aminotransferase abnormalities—this admission completed genetic confirmation (Figure 4). Whole-exome sequencing identified a DMD variant, NM_004006.3:c.1351G>T (p.Asp451Tyr); Sanger sequencing confirmed hemizygosity in the proband, while his mother was heterozygous, consistent with an X-linked recessive inheritance pattern. Family inquiry further revealed neuromuscular phenotypes among maternal relatives resembling muscle weakness/atrophy (with some progressing to severe functional limitation), supporting an association between the identified variant and the family phenotype (Figure 5) (6).

**Figure 4:**
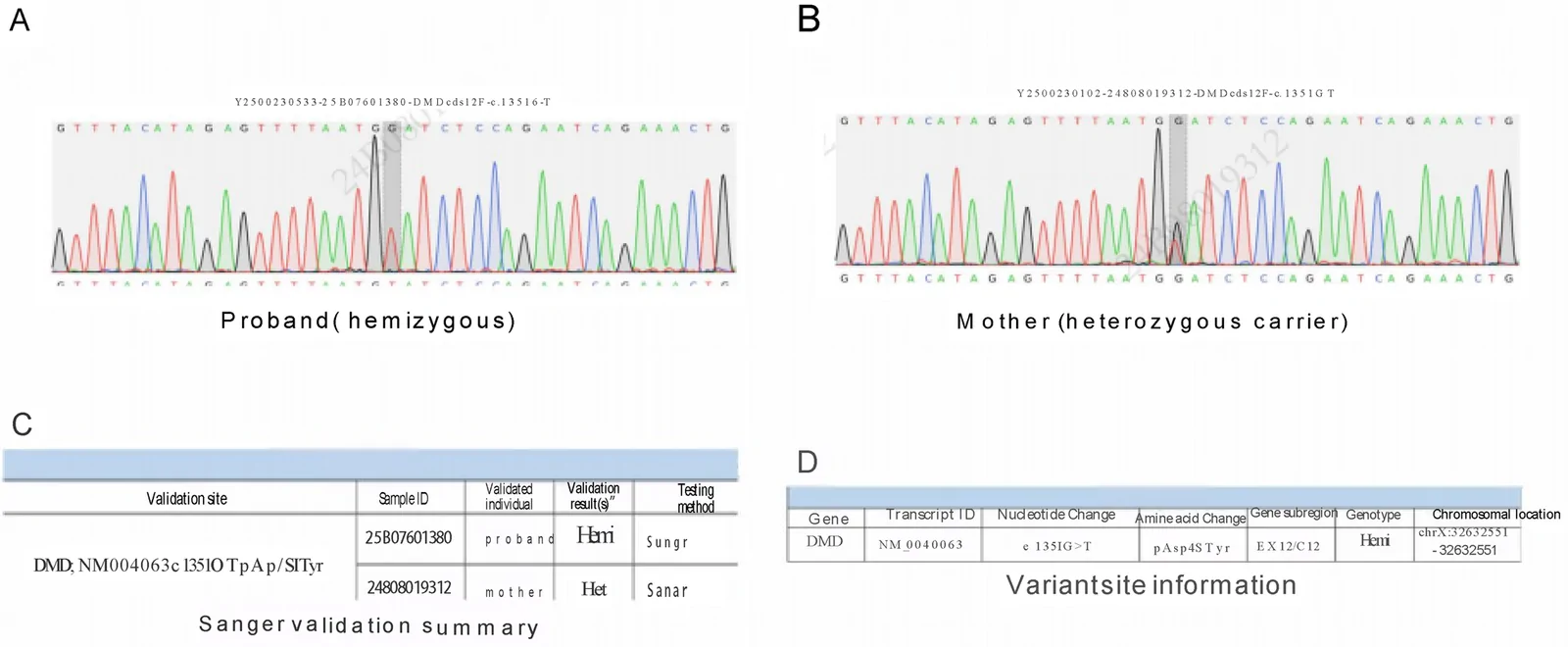
Genetic testing results. **(A)** Sanger sequencing chromatogram of the proband, demonstrating the hemizygous DMD variant NM_004006.3:c.1351G>T (p.Asp451Tyr). **(B)** Sanger sequencing chromatogram of the patient's mother, demonstrating heterozygous carrier status for the same variant. **(C)** Summary of the Sanger validation results for the proband and his mother. **(D)** Variant-site information, including the gene, transcript, nucleotide change, amino-acid change, gene subregion, genotype, and chromosomal location.

**Figure 5:**
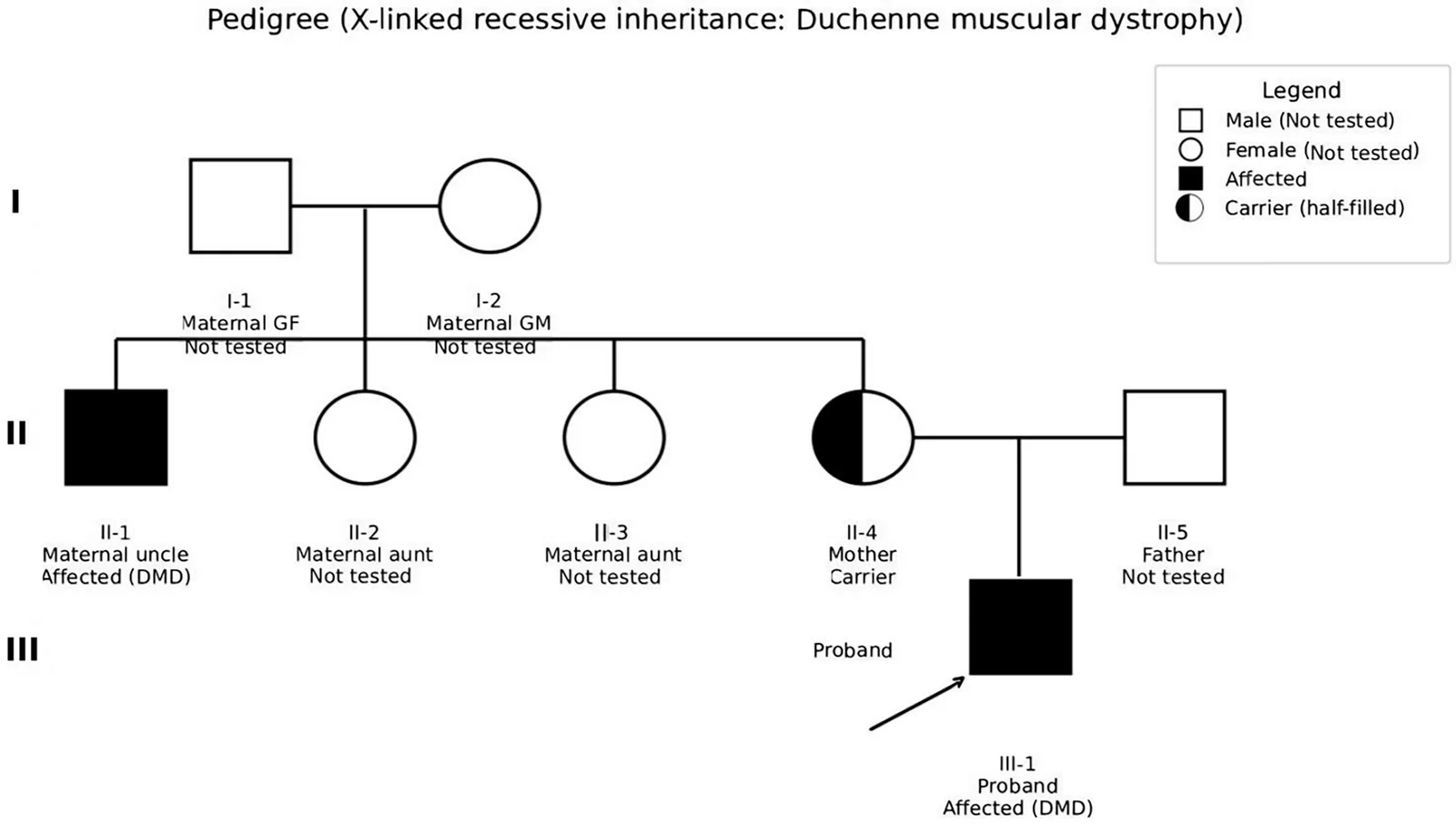
Pedigree of the family with X-linked recessive Duchenne muscular dystrophy. Squares indicate males and circles indicate females. The proband is marked with an arrow. Filled symbols indicate clinically affected individuals with Duchenne muscular dystrophy, and half-filled circles indicate confirmed female carriers. “Not tested/unknown” denotes relatives without genetic testing, regardless of whether they were clinically asymptomatic. Where available, genetic testing status is annotated as WES plus Sanger confirmed, Sanger confirmed, tested negative, or not tested. Clinically asymptomatic status should not be interpreted as genetically unaffected.

### 2.5 Follow-up

Following discharge after the initial presentation, the patient remained on guideline-directed medical therapy for heart failure, and his cardiopulmonary symptoms demonstrated a sustained, stepwise improvement over time. Objective documentation of cardiac functional recovery was available at each major clinical milestone. During the second hospitalization, left ventricular systolic function was reassessed by transthoracic echocardiography on 2025-11-12, which reported an LVEF of 50% (the corresponding source is specified in Table 1 as “echo report, Nov 2025”). For the third hospitalization, we subsequently retrieved the contemporaneous transthoracic echocardiography report from the institutional system, confirming a further increase in LVEF to 55%. Taken together, these serial echocardiographic data allow a clear longitudinal depiction of cardiac function across the three admissions—approximately 44% at the first hospitalization (Sep 2025), improving to 50% at the second hospitalization (Nov 2025), and further to 55% at the third hospitalization (Dec 2025)—which is concordant with the observed clinical stabilization under continued cardioprotective therapy and provides the quantitative basis for the treatment-response trajectory summarized in (Table 1). Ambulatory Holter ECG monitoring was not performed during the reported hospitalization period; therefore, clinically silent arrhythmias cannot be excluded. Given the recognized arrhythmic risk in DMD-associated cardiomyopathy, annual Holter monitoring was recommended as part of the post-discharge follow-up plan.

**Table 1:** Longitudinal tracking of key clinical indicators.

Variables	2025-05-01 (outside hospital/prior history)	First hospitalization (2025-09-16 to 2025-09-24)	Second hospitalization (2025-11-11 to 2025-11-19)	Third hospitalization (2025-12-10 to 2025-12-23)
Chief complaint/key symptoms	Chest tightness for 2 years, worsening over the past 4 months	Chest tightness for 2 years with worsening over the past 5 months; fatigue and reduced exercise tolerance, with occasional palpitations/chest pain	Limb weakness for >4 years; intermittent chest tightness and dyspnea	Marked improvement in heart failure symptoms
NYHA class (estimated)	III	II	II	I-II
Cardiac structure and function				
LVEF (%)	40	44	50	55
LV EDV (mL)	NR	∼135	NR	NR
LVEDVi	NR	NR	NR	NR
LV mass	NR	NR	NR	NR
LA size	NR	enlarged	NR	NR
RA size	NR	NR	NR	NR
RV systolic function	NR	preserved	NR	NR
TAPSE (cm)	NR	∼2.2	NR	NR
PASP (mmHg)	NR	∼23	NR	NR
Clinical CMR report	not performed	impaired LV systolic function; multifocal patchy LGE, predominantly subepicardial; trabeculated/noncompaction-like LV morphology	not performed	not performed
Strain	NR	NR	NR	NR
Mapping values	NR	NR	NR	NR
Holter ECG	not performed	not performed	not performed	not performed
Coronary CTA	/	No significant coronary stenosis; superficial myocardial bridging in the mid-LAD	/	/
hs-cTnT (µg/L)	/	0.038	0.033	0.038
NT-proBNP (pg/mL)	/	21.45	25.35	33.1
Skeletal muscle/neurophysiology				
Electromyography (EMG)	/	Myopathic changes with widespread degenerative muscle fiber alterations; no evidence of nerve conduction block	/	/
Myoglobin (µg/L)	206	205	/	/
CK (U/L)	/	1,872	1,451	745
CK-MB (U/L)	/	32	23	23
Metabolic/renal/hepatic function				
HbA1c (%)	8	8.5	/	7.7
Fasting plasma glucose (mmol/L)	10.13	8.72	7.9	7.9
24-hour urinary protein (mg/L)	923	649	/	319
UACR (mg/g)	/	/	/	389.08
ALT (U/L)	186.3	132	102	121
AST (U/L)	138.9	72	48	61
Key therapies (discharge/maintenance focus)				
ACEI/ARB	/	Sacubitril/valsartan 100 mg twice daily	maintenance	maintenance
β-blocker	Bisoprolol (dose not specified)	Bisoprolol → metoprolol succinate 47.5 mg once daily	maintenance	maintenance
SGLT2i	/	Dapagliflozin 10 mg once daily	maintenance	maintenance
MRA	/	Spironolactone 20 mg once daily	Switched to finerenone 20 mg once daily	finerenone 20 mg once daily (continued)
Loop diuretic	/	Furosemide 20 mg once daily	Discontinued	Discontinued
Glucocorticoids	/	/	Added: Prednisone 30 mg once daily	Prednisone 30 mg once daily (continued)
Glucose-lowering regimen	/	Metformin 500 mg twice daily	Added: acarbose 100 mg three times daily	Added: semaglutide 0.25 mg once weekly + voglibose 0.2 mg once daily

NR, not reported or not retrospectively available from the archived clinical records. Feature-tracking strain analysis, mapping values, indexed volumes, and LV mass were not available from the original clinical CMR acquisition/report. CMR findings were extracted from the original clinical imaging report.

## 3 Patient perspective

“I had chest tightness and felt unusually tired for a long time, and over the past months it became harder for me to exercise or even walk uphill. Before the diagnosis was clear, I was worried because I kept coming back to the hospital and didn't understand why my heart function was reduced. After the doctors explained that my heart problem was related to Duchenne muscular dystrophy, my family and I felt we finally had an answer and a clearer plan. With the medications and follow-up, my symptoms improved and I can do more daily activities than before. We also understand why my mother needs screening as a carrier, and why our family members may need evaluation. We hope this report helps other patients like me get the right tests earlier and avoid delays in diagnosis.”

## 4 Discussion

### 4.1 Diagnostic pitfalls and clinical clues

The key lesson from this case is that when a 17-year-old adolescent male first presents with heart failure or a dilated cardiomyopathy–like phenotype, an evaluation confined to a “single-system cardiology pathway” can easily miss an underlying dystrophinopathy. At the first hospitalization (September 2025), the patient already had left ventricular enlargement with an LVEF of approximately 44%, accompanied by markedly elevated creatine kinase (1,451 U/L) and increased aminotransferases; this constellation should prompt early inclusion of “inherited myopathy–related cardiac involvement” in the differential diagnosis and proactive questioning for clues such as progressive proximal weakness and difficulty rising from a chair or climbing stairs (1).

During the second hospitalization, bedside examination identified classic neuromuscular signs—including a positive Gower's sign, calf pseudohypertrophy, and a waddling gait—and electromyography confirmed a myopathic pattern, allowing “cardiac involvement + skeletal muscle involvement” to be unified within a single etiologic chain. This cross-system integration substantially shortened the diagnostic trajectory from “non-ischemic cardiomyopathy” to “DMD-associated cardiomyopathy”. In similar patients, a practical “red-flag cluster” can improve diagnostic sensitivity: adolescent male + unexplained DCM/heart failure + elevated CK and/or (myogenic) aminotransferase elevation + weakness or gait abnormality, with early referral to an integrated neuromuscular–cardiac evaluation and genetic testing (1).

This case also highlights a practical pitfall: even when key information is present in the record (e.g., maternal family history of DMD and progressive weakness), it may be underweighted if the assessment proceeds along a single-system cardiology pathway. Systematically prioritizing these “red flags” at first presentation can substantially shorten the diagnostic interval by prompting early CK screening, neuromuscular consultation, EMG, and genetic testing. Importantly, while the acute heart failure treatment would remain anchored in guideline-directed medical therapy, earlier etiologic recognition enables timely transition to integrated neuromuscular–cardiac care, anticipatory surveillance, and proactive genetic counseling and carrier screening.

### 4.2 Cardiac phenotype in DMD and role of CMR

In this case, echocardiography established left ventricular enlargement and reduced systolic function, while coronary CTA excluded significant obstructive coronary disease. The original clinical CMR report additionally described multifocal patchy LGE with a predominantly subepicardial distribution and a trabeculated/noncompaction-like left ventricular morphology. Because a complete publication-quality CMR image set was not available for independent visual presentation, these findings were treated as report-based supportive information rather than as standalone imaging proof. The etiologic diagnosis was instead established through convergence of the cardiac phenotype with marked hyperCKemia, progressive proximal muscle weakness, characteristic neuromuscular signs, a myopathic EMG pattern, maternal family history, and confirmatory DMD genetic testing.

More broadly, CMR remains clinically valuable in DMD because LGE can identify myocardial fibrosis before overt deterioration in conventional systolic indices and may assist with risk stratification and longitudinal surveillance. However, imaging findings should be interpreted within the full clinical and genetic context, particularly when technical parameters, complete sequences, or serial examinations are unavailable. DMD patients may also experience acute myocardial injury or myocarditis-like episodes that can mimic acute coronary syndromes, underscoring the need to interpret chest pain, troponin elevation, and imaging findings in the context of the underlying dystrophinopathy rather than relying on a single imaging feature (9, 10).

### 4.3 Therapeutic considerations and response

The therapeutic strategy in this case followed two parallel tracks: sustained cardioprotection anchored in guideline-directed medical therapy (GDMT) for heart failure, alongside timely initiation of multidisciplinary management tailored to DMD. During the first hospitalization, the patient was started on a comprehensive HF regimen centered on an ARNI, a β-blocker, an MRA, an SGLT2 inhibitor, and diuretics as needed for congestion control. Across the second and third hospitalizations and subsequent follow-up, his LVEF improved longitudinally from ∼44% to 50% and further to 55%, suggesting that, in real-world practice, early establishment and continued adherence to a cardioprotective strategy may be associated with functional improvement or stabilization (6). Prior studies support the potential benefit of neurohormonal blockade—particularly ACEI/ARB and β-blockers—in DMD-associated cardiac involvement (7), and randomized controlled evidence further suggests that introducing an MRA (e.g., eplerenone) at an early stage may attenuate the trajectory of LV functional deterioration (8). Building on this framework, once neuromuscular clues became evident, glucocorticoid therapy was initiated with structured monitoring for bone and metabolic complications. Notably, the third hospitalization was driven by dysglycemia and proteinuria, underscoring the intertwined “cardiac–metabolic–renal–skeletal muscle” burden in DMD and reinforcing the need for coordinated multidisciplinary follow-up rather than a single-organ management approach (2).

The patient was admitted for the third time on 10 December 2025 (hospital stay: 13 days) because of hyperglycemia with proteinuria of approximately 3 months’ duration, with the primary goal of evaluating and treating type 2 diabetes mellitus with renal involvement. By this time, a dystrophinopathy with cardiac involvement had been strongly established by the evolving clinical course, and heart-failure–related symptoms were relatively mild. On examination, his BMI was ∼31.8 kg/m^2^ and there was no clinically evident peripheral edema; functional status was estimated as NYHA class I–II (estimated). A key contextual consideration for this admission was the patient's high cardiometabolic vulnerability in the setting of DMD, where progressive skeletal muscle loss (reducing glucose disposal capacity), limited physical activity, and increased adiposity can converge to promote insulin resistance and downstream cardio–renal risk. In addition, once glucocorticoid therapy is initiated for DMD management, glycemic deterioration may occur or accelerate, further increasing the likelihood of diabetes-related complications and necessitating structured monitoring and early intervention.

Investigations during this admission included: (1) ECG showing sinus rhythm/sinus arrhythmia, with abnormal Q waves in leads III and aVF and T-wave changes in leads I and aVL, to be interpreted in the context of prior CMR/LGE findings; (2) transthoracic echocardiography showing further improvement in LV systolic function with LVEF ∼55%; (3) longitudinal cardiac biomarkers indicating persistently mild myocardial injury with low congestion burden, alongside ongoing skeletal muscle injury markers; and (4) metabolic–renal profiling confirming dysglycemia and albuminuric kidney involvement, with preserved renal function (creatinine ∼46 μmol/L). Aminotransferases remained elevated, consistent with the established pattern of myogenic enzyme elevation in this patient.

Management during this hospitalization primarily targeted the metabolic–renal axis, while continuing stable cardioprotective therapy and DMD-directed care. Heart failure/cardiomyopathy management was maintained with sacubitril/valsartan, a β-blocker (metoprolol succinate), an SGLT2 inhibitor (dapagliflozin), and an MRA (spironolactone), with the addition or substitution of finerenone considered for renal/albuminuria protection as documented in the medical record. No evidence suggested a need for intensified diuresis or acute decompensated HF rescue therapy during this admission. This cardioprotective strategy aligns with contemporary long-term management principles for DMD-associated cardiomyopathy (6, 7). For glycemic and weight management, semaglutide was initiated at 0.25 mg weekly in addition to lifestyle counseling and background oral therapy. Given the patient's neuromuscular disease context, particular attention was paid to tolerability and safety—especially gastrointestinal adverse effects that could precipitate reduced intake or dehydration (relevant in patients receiving cardiometabolic agents), as well as the need to avoid disproportionate loss of lean mass during weight reduction by integrating nutrition and rehabilitation planning into follow-up.

The patient remained clinically stable throughout hospitalization, with further symptom improvement and no HF deterioration. He was discharged after 13 days with a plan for integrated follow-up, including repeat echocardiography, Holter monitoring for arrhythmia surveillance, and consideration of repeat CMR for risk stratification; pulmonary function testing and formal functional scales were not assessed during hospitalization and were therefore scheduled as priorities during longitudinal follow-up. During this admission, the diagnostic loop was completed at the molecular level: WES identified a DMD variant (NM_004006.3:c.1351G>T; p.Asp451Tyr), and Sanger sequencing confirmed hemizygosity in the proband and heterozygosity in his mother (Figure 4), consistent with X-linked recessive inheritance. The family pedigree and available testing status are summarized in (Figure 5): WES identified a DMD variant (NM_004006.3:c.1351G>T; p.Asp451Tyr), and Sanger sequencing confirmed hemizygosity in the proband and heterozygosity in his mother, consistent with X-linked recessive inheritance. Family inquiry suggested neuromuscular phenotypes among maternal relatives, supporting segregation with the family phenotype (6).

### 4.4 Genetic counseling and carrier surveillance

In this case, genetic testing provided the decisive “closure” of the diagnostic loop. Whole-exome sequencing (WES) identified a DMD variant (NM_004006.3:c.1351G>T; p.Asp451Tyr), which was subsequently confirmed by Sanger sequencing as hemizygous in the proband and heterozygous in his mother—fully consistent with X-linked recessive inheritance. Together with the family history indicating neuromuscular phenotypes among maternal relatives, these findings further supported the etiologic relevance of the variant. Clinically, this type of closed-loop reasoning—moving from phenotypic clues to electrophysiologic/imaging phenotypes, then to molecular evidence and familial co-segregation—is particularly valuable. It not only consolidates previously disparate signals (heart failure, weakness signs, myopathic injury, and characteristic CMR findings) into a single etiologic chain, but also strengthens the interpretability and reproducibility of the diagnostic conclusion. Importantly, genetic confirmation expands the management focus from “explaining the current presentation” to “anticipating foreseeable risks,” enabling follow-up to transition from reactive symptom-driven care to proactive surveillance and timely intervention grounded in a defined genetic risk profile. Regarding additional confirmatory testing, muscle biopsy was not performed because a molecular diagnosis was established with genotype–phenotype coherence, and biopsy was unlikely to alter near-term management while adding procedural burden. Similarly, MLPA or other targeted assays for exon-level deletions/duplications were not pursued, as the WES workflow included CNV calling and did not identify phenotype-relevant pathogenic/likely pathogenic chromosomal CNVs, making a clinically significant large structural event less likely in this patient; nevertheless, MLPA (or another orthogonal copy-number method) remains appropriate when sequencing is negative/inconclusive or when a structural variant is strongly suspected despite non-diagnostic initial testing.

The family-level implications extend beyond etiologic attribution for the proband to recognition and longitudinal surveillance of cardiac involvement in female carriers. Although the patient's mother had no overt weakness, as a carrier she should be enrolled in periodic cardiac screening (ECG and echocardiography, with CMR when indicated) and undergo risk stratification before symptoms emerge (6). In addition to the proband's mother, we would recommend genetic counseling and carrier testing for potentially at-risk female relatives in the maternal lineage (e.g., II-2 and II-3), and baseline cardiac screening (ECG and echocardiography, with CMR when indicated), given the possibility of carrier status and the recognized risk of subclinical cardiac involvement in carriers. In practice, this point is frequently underestimated: skeletal muscle manifestations in female carriers may be subtle or absent, fostering the misconception that “asymptomatic” individuals do not require follow-up. Yet myocardial involvement can evolve subclinically, with cumulative risk increasing with age. Accordingly, the core aims of carrier surveillance are early detection, early stratification, and early action. A practical approach is to establish ECG and echocardiography as baseline assessments, escalating to CMR when structural/functional abnormalities, arrhythmic signals, or equivocal echocardiographic findings arise, thereby enabling characterization of fibrosis burden and distribution. This information can then guide surveillance intervals, lifestyle counseling, and—when appropriate—consideration of earlier cardioprotective therapy (6). From a communication standpoint, carriers should be counseled explicitly that the purpose of screening is not to “look for symptoms,” but to detect actionable early changes while intervention remains most effective, thereby shifting risk control upstream.

Genetic counseling should also cover recurrence-risk assessment and available reproductive options (e.g., prenatal diagnosis and preimplantation genetic testing), while providing an actionable plan for early screening and multidisciplinary follow-up of at-risk relatives, achieving the dual benefit of “patient management + family prevention” (1). In concrete terms, counseling should be oriented toward decision-ready information: clarifying the recurrence-risk framework under X-linked inheritance, outlining the testing pathways and time windows associated with different reproductive choices, and proposing a stratified screening strategy for relatives who have not yet been evaluated (for example, prioritizing clinical screening and, when indicated, genetic testing in maternal male relatives, while offering carrier testing to female relatives in accordance with reproductive plans and individual preferences). For affected males or high-risk individuals, early enrollment into an integrated neuromuscular–cardiac follow-up program is essential, with a longitudinal care plan spanning cardiac function, respiratory status, metabolic risk, and bone health. In this way, the clinical contribution of a single case report can be extended from an isolated patient narrative to a more generalizable model of family-centered risk management (1).

Although both grandparents (Generation I) were clinically unaffected, the presence of multiple affected individuals in Generation II remains compatible with X-linked recessive dystrophinopathy. This can be explained by an asymptomatic female carrier in Generation I (most plausibly the maternal grandmother), germline mosaicism, or a *de novo* event in the maternal lineage. Because genetic testing was not available for Generation I and other maternal relatives in this report, we cannot distinguish among these mechanisms and therefore describe the pedigree as consistent with X-linked inheritance while recommending cascade testing where feasible.

## 5 Conclusion

We report a 17-year-old adolescent male with Duchenne muscular dystrophy (DMD)–associated cardiomyopathy who presented predominantly with chest tightness and reduced exercise tolerance, leading to recurrent hospitalizations. At the first admission, echocardiography already demonstrated left atrial and left ventricular enlargement with impaired systolic function (LVEF ∼44%), accompanied by marked hyperCKemia and mild elevation of myocardial injury biomarkers. Coronary CTA excluded significant obstructive coronary disease, while the original clinical CMR report described a nonischemic myocardial involvement pattern. Electromyography supported a myopathic process, and whole-exome sequencing with Sanger confirmation ultimately established a closed diagnostic loop integrating clinical, imaging, electrophysiologic, and genetic evidence. Under guideline-directed heart failure therapy and multidisciplinary care, cardiac function improved longitudinally (LVEF ∼44% → 50% → 55%). This case underscores that in adolescent males with nonischemic cardiomyopathy and/or heart failure of uncertain etiology—particularly when accompanied by hyperCKemia or suspected “myogenic” aminotransferase elevation—dystrophinopathy should be actively considered. Early integration of cardiac assessment, neuromuscular evaluation, and genetic testing can expedite etiologic clarification, and family-centered management should proceed in parallel, including cardiac surveillance and genetic counseling for female carriers, to optimize long-term outcomes for both the patient and the family.

## Data Availability

The original contributions presented in the study are included in the article/Supplementary Material, further inquiries can be directed to the corresponding author.
